# Supplementary material for: Inhibition of MEK1/2 Signaling Pathway Limits M2 Macrophage Polarization and Interferes in the Dental Socket Repair Process in Mice
Source: Biology (Basel). 2025 Jan 21;14(2):107. doi: 10.3390/biology14020107 (PMC11851886; doi:10.3390/biology14020107)
Supplement: Supplementary file 1 [file biology-14-00107-s001.zip › biology-3285104-supplementary.pdf]

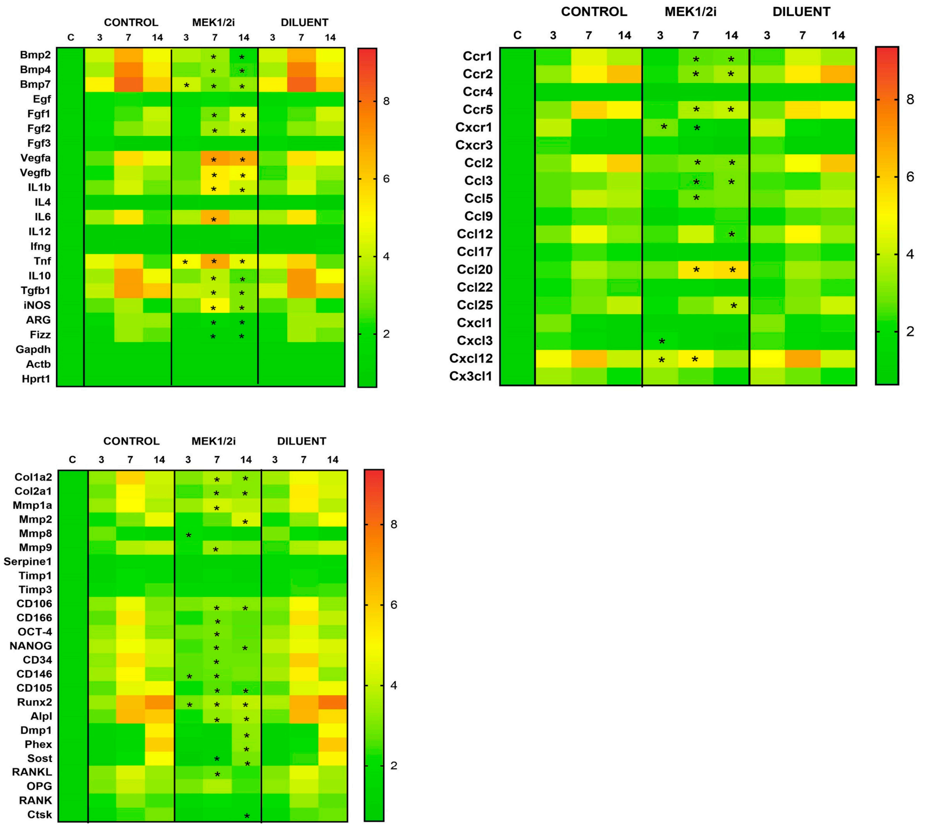

Figure S1 - Comparative Molecular analysis (PCRArray), in the dental socket repair process over the periods of 0, 3, 7 and 14 days after tooth extraction between Control, Diluent and MEK1/2i groups. Using Heat map to quantify the expression of the growth factors (BMPs, TGFβ, VEGFs and FGFs), extracellular matrix markers (COL1a1, COL1a2, MMPs, TIMPs and Serpine), bone markers (RUNX2, DMP1, ALPL, PHEX, SOST, CTSK, RANKL, RANK and OPG), chemokines and their receptors (CCLs, CXCLs; CCRs and CXCRs), mesenchymal stem cell markers (CD34, 105, 106, 116, 146, OCT-4 and NANOG), cytokines (ILs, TNF and IFNG) and markers associated with macrophage polarization (iNOS, ARG, GAPDH, ACTb, HPRT-1 and FIZZ). *($p<0.05$) indicates statistically significant differences between the MEK1/2i versus control group.

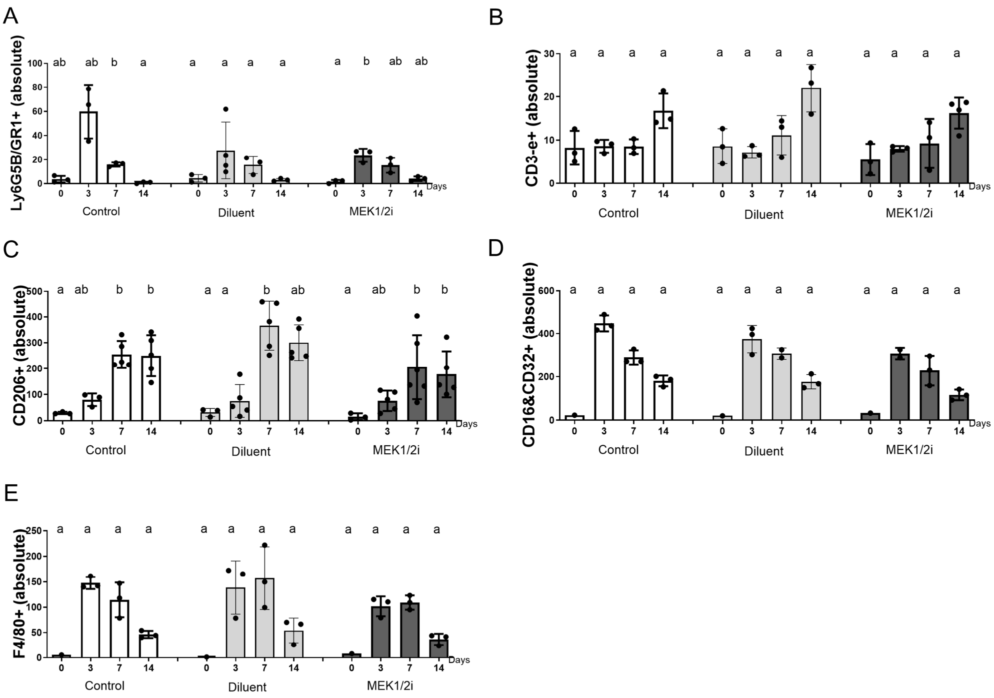

Figure S2 - Comparative IHC analysis, of the quantification of A) Ly6G5B/Gr1+, B) CD3-e+, C) CD206+, D) CD16&CD32+ and E) F4/80+ cells in absolute numbers. Different lowercase letters represent a statistically significant difference (p<0.05) between different time points within the same group; time points within the same group presenting the same letter are not statistically different. *(p<0.05) indicates statistically significant differences between the MEK1/2i versus control group. # (p *(p<0.05) indicates statistically significant differences between the MEK1/2i versus diluent group.

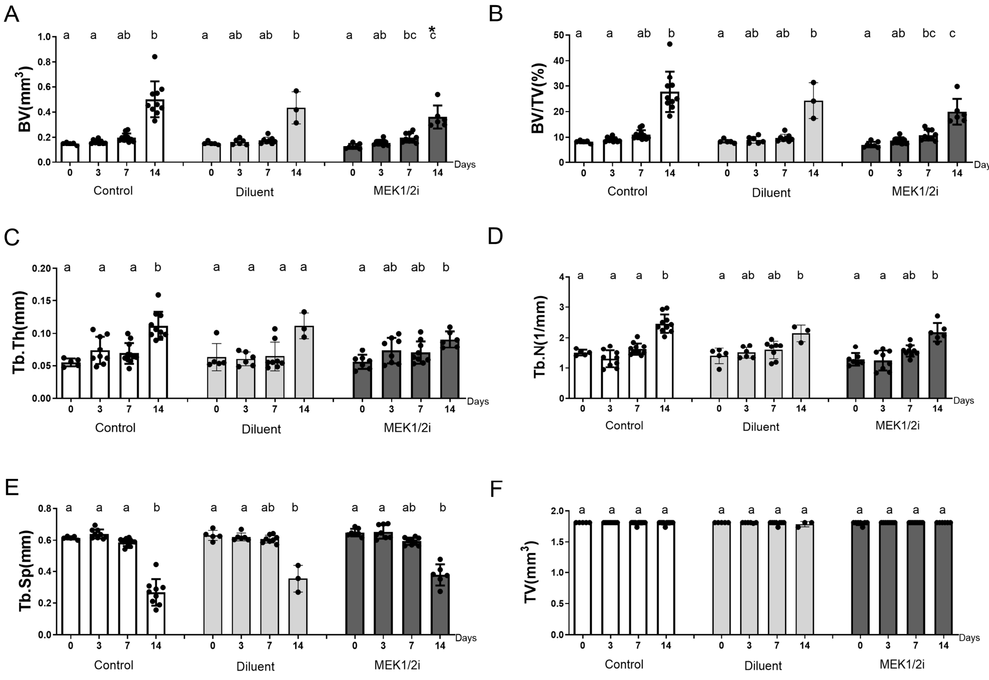

Figure S3 - Comparative uCT analysis, of morphological parameters of bone trabecular microarchitecture in the dental socket repair process over the periods of 0, 3, 7 and 14 days after tooth extraction between Control, Diluent and MEK1/2i groups. Bone trabecular analyzes (A-F) included: A) Bone volume (BV), B) bone fraction in relation to total volume (BV/TV), C), trabecular thickness (Tb.Th), D) number of trabeculae (Tb.N), E) mean distance between trabeculae (Tb.Sp), F) total tissue volume (TV). The results represent the mean and standard deviation values in each of the analyzed periods. Different lowercase letters represent a statistically significant difference ($p<0.05$) between different time points within the same group; time points within the same group presenting the same letter are not statistically different. *($p<0.05$) indicates statistically significant differences between the MEK1/2i versus control group. # (p *($p<0.05$) indicates statistically significant differences between the MEK1/2i versus diluent group.

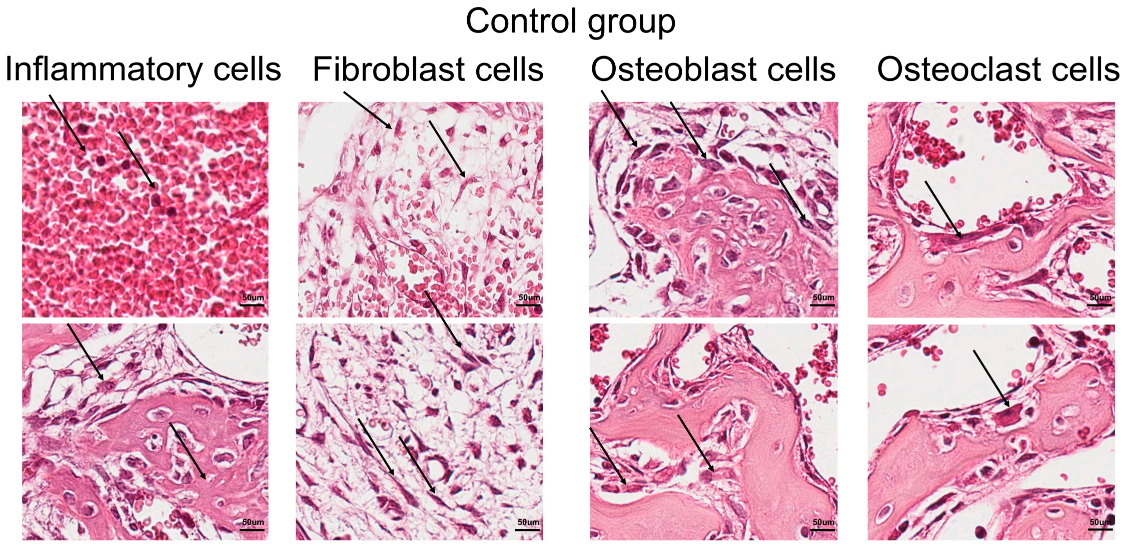

Figure S4 - Photomicrographs are representative of the middle region of the dental socket, and the morphology considered for quantification. HE staining; Scanned on the Aperio Scanscope CS device at 40x objectives; Bar= 50 μm.

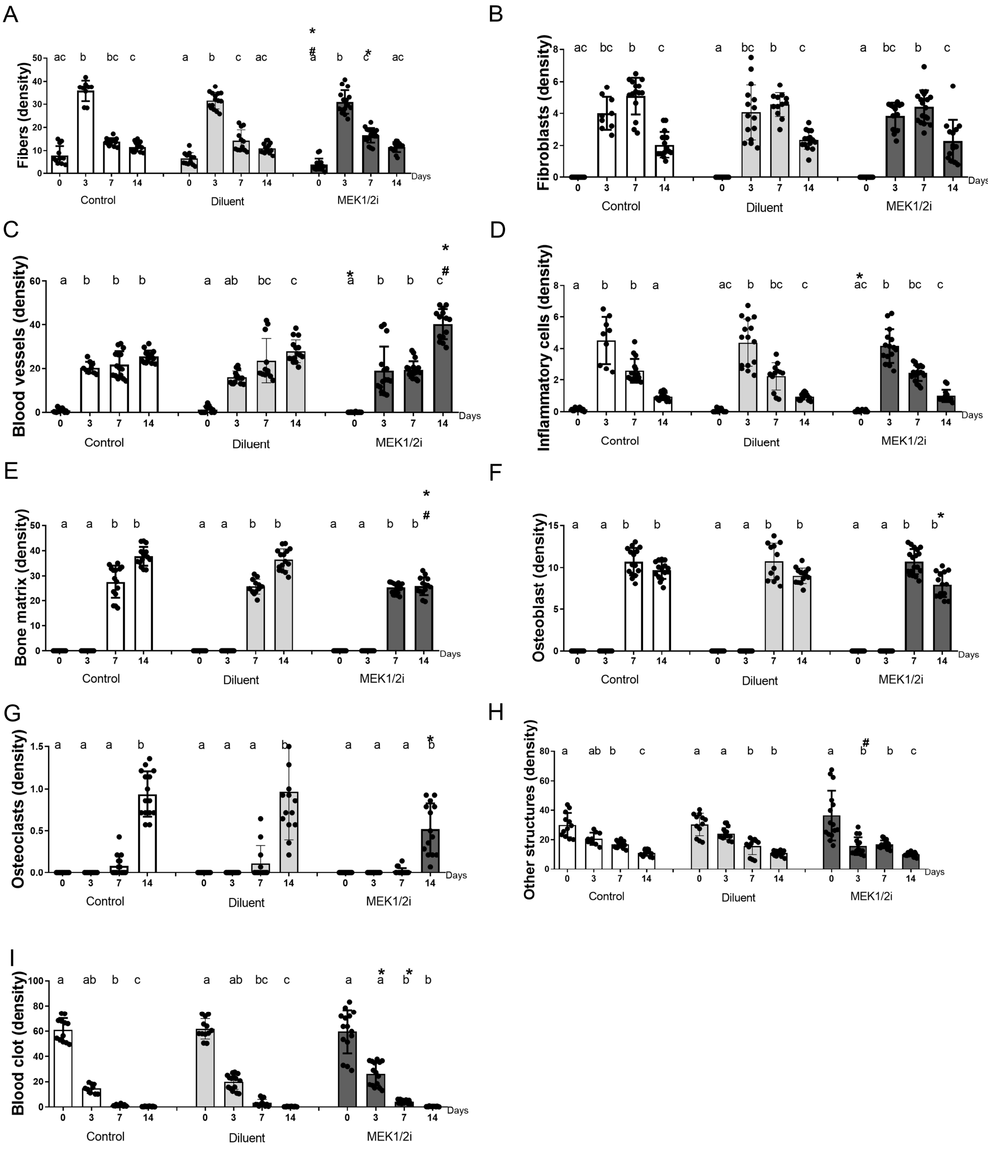

Figure S5 – Comparative histomorphometric analysis, of the total area density (%) occupied by A) collagen fibers, B) fibroblasts, C) blood vessels, D) inflammatory cells, E) bone matrix, F) osteoblasts, G), osteoclasts, H), other structures, I) blood clot in the dental socket repair process over the periods of 0, 3, 7 and 14 days after tooth extraction between Control, Diluent and MEK1/2i groups. The results represent the mean and standard deviation values of the analyzed period. Different lower-case letters represent a statistically significant difference ($p<0.05$) between different time points within the same group; time points within the same group presenting the same letter are not statistically different. *($p<0.05$) indicates statistically significant differences between the MEK1/2i versus control group. # (p *($p<0.05$) indicates statistically significant differences between the MEK1/2i versus diluent group.

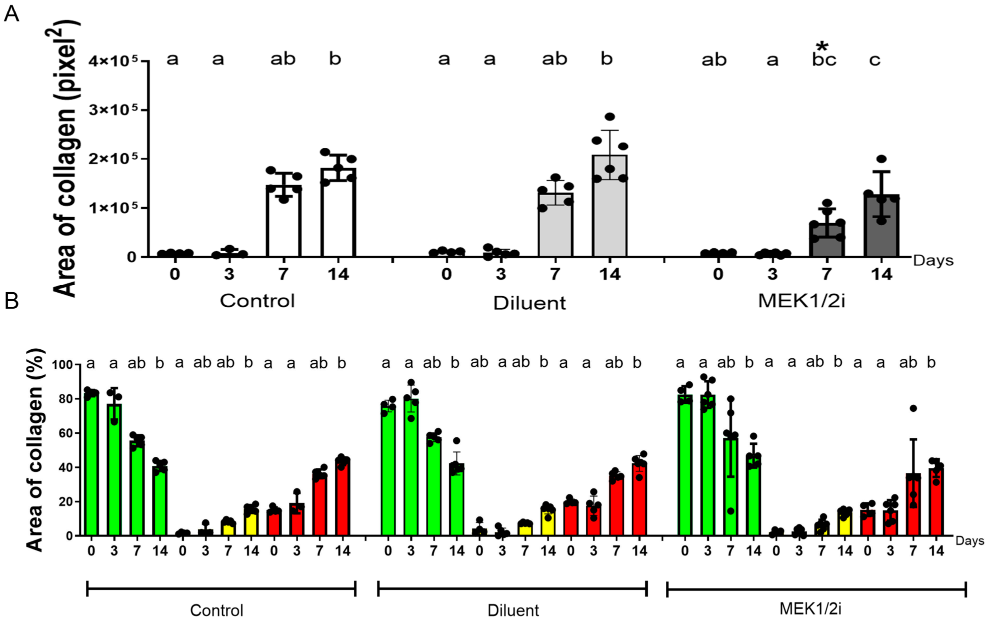

Figure S6 - Comparative birefringence analysis, of the total area of collagen fibers (A - pixel²) and the distinction in percentage of the degree of maturation of collagen fibers in green, yellow, red birefringence color (B - %) in the dental socket repair process over the periods of 0, 3, 7 and 14 days after tooth extraction between Control and MEK1/2i groups. Results are presented as the mean (±SEM) of percentage or pixels² for each color in the birefringence. Different lowercase letters represent a statistically significant difference ($p<0.05$) between different time points within the same group; time points within the same group presenting the same letter are not statistically different. *($p<0.05$) indicates statistically significant differences between the MEK1/2i versus control group.
